# Supplementary material for: The role of land use in terrestrial support of boreal lake food webs
Source: Nat Commun. 2025 Apr 15;16:3572. doi: 10.1038/s41467-025-58505-y (PMC12000567; doi:10.1038/s41467-025-58505-y)
Supplement: Supplementary file 1 — Supplementary Information [file 41467_2025_58505_MOESM1_ESM.pdf]

Supplementary material: "The role of land use in terrestrial support of boreal lake food webs"

Ossi Keva*[1,2], Matthew R. D. Cobain[1], Antti P. Eloranta[1], Heikki Hämäläinen[1], Mikko Kiljunen[1], Jos Schilder[1,3] & Roger I. Jones[1]
*Corresponding author and email: Ossi Keva; ossi.keva@helsinki.fi
[1]Department of Biological and Environmental Science, University of Jyväskylä, P.O. Box 35, FI-40014, Jyväskylä, Finland.
[2]Faculty of Biological and Environmental Science, University of Helsinki, Finland.
[3]Rijkswaterstaat, Ministry of Infrastructure and Water Management, Lelystad, The Netherlands

This supplementary file includes 10 supplementary tables and 9 supplementary figures:

Supplementary Table 1. Environmental variables of the 35 boreal study lakes in Finland

Supplementary Table 2. Variable loadings on the first three PC axes in

Supplementary Table 3. Seasonal and spatial variation in source δ2H values in four lakes

Supplementary Table 4. Sample counts

Supplementary Table 5 Summary table of the sample sizes

Supplementary Table 6. Model slope and intercept posterior results.

Supplementary Table 7. Fish stomach content data (prey proportions in %)

Supplementary Table 8. Comparison of MixSIAR models with differing model structures

Supplementary Table 9. Recalculations of hydrogen isotope omega (ω) values based on the suggestion by Soto et al. (2017, Table 2 equation a) to recalibrate δ2H value of consumers due to bias in earlier standard Supplementary Table 10. Average summertime inlet DOM and POM δ^2H values of the studied lakes

Supplementary Fig. 1. Pairwise correlations between the lake environmental variables

Supplementary Fig. 2. Principal component analysis ordinations

Supplementary Fig. 3. Effect of POM N:C-ratio on consumer allochthony in 35 study lakes

Supplementary Fig. 4. Effect of forest coverage on consumer allochthony in 35 study lakes.

Supplementary Fig. 5. Omega sensitivity analysis for MixSIAR mixing models

Supplementary Fig. 6. Scaled density of linear regression coefficient with PC1 and dietary mixtures in ILR space

Supplementary Fig. 7. Estimated consumer trophic level across the environmental gradient (PC1)

Supplementary Fig. 8. Consumer allochthony models with modelled bacterial δ^2H values as terrestrial source.

Supplementary Fig. 9. Consumer allochthony models with sampled benthic algae δ^2H values as aquatic source.

Supplementary Table 1. Environmental variables of the 35 boreal study lakes in Finland. Lake name abbreviations (abbr.), coordinates in Lat. and Lon., lake morphometrics, catchment area characteristics, chemistry, light environment, and PC1-3 score variables are shown for each lake. Abbreviations of the lake morphometric variables: Lake area (LA), shoreline length (SL), shoreline development index (SD), average depth (Dmean), maximum depth (Dmax), catchment area (CA). Catchment area characteristics are presented as proportions of the catchment (%): urban area (Urb), agricultural area (Agr), forest area (For), wetland area (Wet), and waterbodies (Wat). Abbreviations of the lake chemistry variables: total phosphorous (Tot-P), chlorophyl a (Chl-a), pH, $SUVA_{254}$ index (SUVA), DOM light absorbance slope range ratio (SR) and POM nitrogen carbon-ratio (POM N:C). Principal component analysis scores of the first three components (PC1, PC2, PC3) are shown for each lake, with the variable loadings provided in Table S2.

Lake	Abbr.	Coord. N°, E°	LA (km2)	SL (km)	SD	Dmean (m)	Dmax (m)	CA (km2)	Log(CA:LA)	Urb (%)	Agr (%)	For (%)	Wet (%)	Wat (%)	DOC (mg/L)	Tot-P (µg/L)	Chl-a (µg/L)	pH	SUVA	SR	POM N:C	PC1	PC2	PC3
Alajärvi	ALJ	63.01, 23.87	11.1	33.3	2.5	1.4	7.0	482.0	1.6	3.4	12.2	77.6	2.8	4.0	19.5	62.6	24.7	6.9	13.5	1.3	0.083	-0.24	-2.49	-0.52
Arkusjärvi	ARK	61.53, 29.50	1.1	4.5	1.2	7.9	14.1	3.8	0.5	8.9	12.9	49.2	0.3	28.8	4.5	9.0	3.0	7.6	4.6	0.8	0.113	1.11	3.81	-1.00
Eräjärvi	ERA	61.57, 24.60	8.4	39.6	3.5	2.1	10.0	58.2	0.8	4.0	15.5	64.8	1.6	14.1	7.7	25.8	14.2	7.4	6.9	1.1	0.112	0.94	1.06	-0.89
Haapajärvi	HAP	63.55, 26.99	25.9	87.2	4.1	2.8	8.5	1936.8	1.9	2.2	14.6	75.1	3.2	5.0	14.4	88.1	39.4	7.0	10.9	0.9	0.075	-0.16	-3.67	1.26
Hiidenvesi	HII	60.37, 24.20	29.1	100.9	4.5	6.7	29.4	925.8	1.5	6.8	16.0	67.4	0.8	9.0	8.6	38.3	15.3	7.8	12.4	1.0	0.120	1.53	-0.78	1.91
Hirvijärvi	HIR	62.35, 26.97	1.0	12.8	3.7	1.0	3.0	14.8	1.2	0.3	0.0	90.6	0.7	8.4	17.0	25.5	19.8	6.4	11.9	0.8	0.065	-2.89	-0.84	-1.01
Horkkajärvi	HOR	61.21, 25.16	0.01	0.4	1.6	7.7	12.0	0.4	1.6	0.0	0.0	97.5	0.4	2.1	18.3	17.0	13.8	6.0	11.4	0.8	0.054	-3.58	-1.01	-0.15
Hämeenjärvi	HAM	61.32, 27.26	1.3	16.3	4.0	4.5	17.0	14.4	1.0	1.8	0.7	82.7	0.8	14.0	6.2	7.0	6.0	6.7	8.6	1.0	0.080	-1.49	1.50	-0.07
Joroisselkä	JOR	62.15, 27.91	13.0	23.2	1.6	9.1	55.0	1472.0	2.1	2.9	7.7	71.5	2.7	15.3	10.4	26.0	16.0	7.2	11.2	0.9	0.092	-0.34	-0.62	2.81
Jyväsjärvi	JYV	62.23, 25.75	3.3	14.0	2.0	7.0	25.0	366.9	2.0	15.3	5.7	71.2	0.7	7.1	8.6	25.4	9.5	7.1	9.6	0.8	0.104	-0.12	0.08	0.63
Kakkisenjärvi	KAK	62.74, 27.24	1.9	15.8	3.1	2.1	6.0	25.4	1.1	0.5	0.0	83.6	0.4	15.5	11.8	27.0	16.0	6.2	11.8	0.8	0.060	-2.60	0.28	-0.87
Kakskerranjärvi	KKJ	60.36, 22.24	1.6	15.1	3.2	6.4	15.2	8.8	0.7	13.7	17.4	52.2	0.6	16.0	5.8	19.2	7.7	8.0	5.5	1.3	0.103	2.76	1.93	-0.95
Kuhajärvi	KUH	65.92, 26.69	3.1	12.2	1.9	2.1	6.3	16.0	0.7	3.1	5.3	57.4	5.5	28.7	9.3	39.0	27.6	7.0	8.6	0.9	0.148	0.55	1.67	-1.66
Kuontijärvi	KUO	66.15, 29.01	6.0	25.5	2.7	1.7	12.9	89.1	1.2	3.7	5.2	69.3	6.2	15.5	6.8	24.0	12.0	7.5	7.3	1.0	0.096	-0.11	1.05	-0.62
Köyliönjärvi	KOY	61.10, 22.36	12.4	36.8	2.6	2.6	12.8	145.2	1.1	8.5	27.7	53.6	2.0	8.2	7.2	112.4	72.2	8.4	4.9	1.4	0.126	4.77	-1.35	-2.17
Majajärvi	MAJ	61.22, 25.14	0.03	1.1	2.1	4.0	12.0	1.9	1.8	0.0	0.0	96.4	0.2	3.4	17.3	32.0	6.0	5.8	12.5	0.9	0.063	-3.56	-1.29	-0.24
Niemisjärvi	NIE	63.65, 26.48	4.2	28.6	3.7	1.6	5.0	181.1	1.6	2.5	19.6	73.2	1.9	2.9	24.3	66.3	36.9	7.0	11.2	0.8	0.087	-0.83	-3.07	-1.01
Pankajärvi	PAN	61.35, 26.60	5.0	34.6	4.0	3.3	10.0	206.1	1.6	2.1	3.9	71.0	0.6	22.4	13.3	19.0	29.0	7.1	11.2	0.9	0.082	-1.03	0.05	-0.38
Pesosjärvi	PES	66.30, 29.51	0.4	3.9	1.7	4.7	11.9	5.9	1.1	0.1	0.0	86.3	3.9	9.7	6.2	5.5	1.8	7.4	7.6	1.0	0.066	-1.65	1.39	-0.38
Pusulanjärvi	PUS	60.46, 23.98	2.1	9.0	1.7	4.9	10.6	223.4	2.0	5.4	15.4	69.9	0.6	8.8	6.5	47.2	25.7	7.5	11.8	0.9	0.130	0.89	-0.86	-0.35
Pyhäjärvi	PYH	60.72, 26.00	13.0	28.8	2.0	21.1	68.0	457.7	1.5	4.2	30.2	59.1	0.5	5.9	7.6	57.8	20.8	8.0	18.3	1.1	0.193	3.98	-0.98	3.39
Pääjärvi	PAA	61.06, 25.14	13.4	36.6	2.5	14.8	85.0	223.8	1.2	3.0	15.3	73.5	0.6	7.6	9.8	11.3	5.5	7.3	12.1	0.9	0.107	0.23	1.03	3.40
Ruokojärvi	RUO	61.64, 28.40	1.3	12.8	3.2	4.7	24.1	6.2	0.7	0.5	1.8	76.1	1.0	20.6	6.3	5.0	2.7	7.1	8.1	1.0	0.084	-0.91	2.50	-0.16
Suuri Jukajärvi	SUJ	61.52, 28.90	3.6	12.6	1.8	23.0	49.8	56.6	1.2	2.1	1.0	77.1	2.0	17.8	6.3	4.7	3.4	7.2	7.8	1.0	0.077	-1.37	1.99	-0.18
Suuri-Vahvanen	SUU	61.68, 27.55	1.3	14.2	3.4	4.1	15.0	7.0	0.7	2.5	0.4	72.7	0.5	23.9	5.9	3.9	2.1	7.0	18.8	0.8	0.080	-0.55	2.64	2.40
Sääksjärvi	SAJ	62.17, 25.73	0.6	3.6	1.4	5.6	16.1	6.1	1.0	30.2	0.0	59.8	0.3	9.7	6.3	7.0	4.7	7.1	13.7	1.0	0.094	0.77	2.03	-0.69
Sääskjärvi	SAA	60.83, 26.22	5.1	12.1	1.4	2.4	5.0	65.1	1.1	3.8	33.2	55.1	0.2	7.7	6.1	103.2	28.8	7.7	15.3	1.2	0.154	3.49	-1.23	-1.50
Tottijärvi	TOT	61.40, 23.33	0.7	4.8	1.7	2.4	5.7	5.6	0.9	12.6	22.7	50.7	0.9	13.2	6.7	47.3	50.6	8.0	10.2	1.0	0.130	3.08	-0.02	-2.26
Valkea-Kotinen	VAL	61.24, 25.06	0.04	1.0	1.6	3.5	6.5	0.2	0.7	0.0	0.0	80.0	0.0	20.3	13.3	22.3	14.4	5.5	12.3	0.8	0.043	-3.06	1.04	-1.35
Vesijärvi	VES	61.02, 25.61	107.5	227.3	4.9	6.1	40.0	510.1	0.7	8.6	18.0	51.1	1.7	20.6	5.0	31.0	5.0	7.6	7.4	1.1	0.124	3.13	2.05	2.81
Viitaanjärvi	VIT	62.51, 27.03	3.6	28.8	4.0	3.9	14.7	1334.6	2.6	1.8	8.0	82.2	3.5	4.3	12.3	46.5	16.8	6.7	12.5	1.0	0.056	-2.16	0.20	-0.92
Viippero	VIP	63.58, 27.32	1.0	7.7	2.2	3.3	10.0	10.2	1.0	0.6	0.1	88.2	0.3	10.9	11.1	56.9	10.0	6.5	10.4	0.9	0.057	-1.66	-2.85	1.53
Villikkalanjärvi	VIL	60.77, 26.04	7.2	14.0	1.3	2.9	8.9	411.5	1.8	4.1	30.8	61.7	0.3	3.0	8.5	120.4	22.6	7.6	16.5	0.8	0.163	2.37	-2.60	-0.47
Ylisjärvi	YLI	60.36, 23.28	1.8	9.1	1.9	2.1	4.3	129.1	1.9	5.6	24.5	65.0	0.7	4.2	8.4	114.2	30.8	7.5	8.0	1.0	0.141	2.10	-1.71	-1.41
Älänne	ALA	63.49, 28.13	10.0	46.7	3.7	3.2	15.4	357.6	1.6	0.5	0.5	82.0	8.5	8.5	10.2	21.0	11.0	5.5	14.9	0.8	0.058	-3.25	-1.02	0.79

Supplementary Table 2. Variable loadings on the first three PC axes in the principal component analysis, with the most important variables in PC1 highlighted in bold.

Variable	PC1	PC2	PC3
MeanD	0.072	0.163	0.472
MaxD	0.091	0.125	0.565
LA	0.151	0.017	0.315
CA	0.031	-0.295	0.362
Log(CA:LA)	-0.068	-0.424	0.214
SD-index	-0.004	-0.075	0.218
**Urb (%)**	**0.224**	0.135	-0.073
**Agr (%)**	**0.389**	-0.216	-0.006
**For (%)**	**-0.405**	-0.129	0.066
Wet (%)	-0.073	-0.057	0.060
Wat (¤)	0.005	0.441	-0.066
**Tot-P**	**0.297**	-0.353	-0.131
**Chl-a**	**0.203**	-0.323	-0.259
**DOC**	**-0.238**	-0.344	-0.055
**pH**	**0.407**	0.064	0.025
SUVA-254	-0.070	-0.229	0.165
**SR-ratio**	**0.275**	0.026	-0.036
**POM N:C**	**0.394**	-0.028	0.028

Supplementary Table 3. Seasonal and spatial variation in source δ^2H values in four lakes. The grand SD values of inlet DOM and benthic algae were used in MixSIAR models for all the 35 study lakes.

Material	Lake	Season	n	mean	SD
inlet DOM	Jyväsjärvi	Fall	2	-107.3	8.1
inlet DOM	Jyväsjärvi	Spring	3	-111.1	16.6
inlet DOM	Jyväsjärvi	Summer	2	-124.1	8.7
inlet DOM	Jyväsjärvi	Winter	2	-106.3	2.3
inlet DOM	Pääjärvi	Fall	1	-98.1	*NA*
inlet DOM	Pääjärvi	Spring	2	-113.4	13.0
inlet DOM	Pääjärvi	Summer	1	-120.0	*NA*
inlet DOM	Pääjärvi	Winter	2	-113.5	10.8
inlet DOM	Ristinselkä	Summer	1	-128.5	*NA*
inlet DOM	Sääksjärvi	Fall	2	-112.9	8.3
inlet DOM	Sääksjärvi	Spring	3	-106.5	13.9
inlet DOM	Sääksjärvi	Summer	3	-117.5	16.1
inlet DOM	Sääksjärvi	Winter	2	-111.3	8.3
**Grand mean (weighed average)**			**26**	**-112.7**	**11.2**
Benthic algae	Jyväsjärvi	Fall	1	-154.7	*NA*
Benthic algae	Jyväsjärvi	Spring	2	-237.7	5.5
Benthic algae	Jyväsjärvi	Summer	5	-194.8	37.8
Benthic algae	Pääjärvi	Fall	1	-179.0	*NA*
Benthic algae	Pääjärvi	Spring	1	-174.8	*NA*
Benthic algae	Pääjärvi	Summer	2	-221.1	8.4
Benthic algae	Ristinselkä	Summer	6	-207.5	21.8
Benthic algae	Sääksjärvi	Fall	1	-161.8	*NA*
Benthic algae	Sääksjärvi	Spring	1	-155.7	*NA*
Benthic algae	Sääksjärvi	Summer	4	-175.1	25.0
**Grand mean (weighed average)**			**24**	**-194.3**	**23.6**
Lake water	Jyväsjärvi	Fall	2	-79.4	1.0
Lake water	Jyväsjärvi	Spring	2	-86.7	5.6
Lake water	Jyväsjärvi	Summer	2	-83.5	4.1
Lake water	Jyväsjärvi	Winter	2	-83.1	1.5
Lake water	Pääjärvi	Fall	1	-75.4	*NA*
Lake water	Pääjärvi	Spring	1	-78.2	*NA*
Lake water	Pääjärvi	Summer	1	-74.5	*NA*
Lake water	Pääjärvi	Winter	1	-78.3	*NA*
Lake water	Ristinselkä	Summer	7	-76.0	0.7
Lake water	Sääksjärvi	Fall	2	-75.9	0.0
Lake water	Sääksjärvi	Spring	2	-79.8	3.3
Lake water	Sääksjärvi	Summer	3	-78.0	1.7
Lake water	Sääksjärvi	Winter	2	-78.9	1.0
**Grand mean (weighed average)**			**28**	**-78.8**	**1.8**

Supplementary Table 4. Sample counts. The number of analyzed consumer (columns) samples are shown for each lake (rows). The row and column summaries are highlighted in bold.

**Lake**	**Bulk_ZPL**	**Chaoborus**	**Cladocera**	**Copepods**	**Bulk_BMI littoral**	**Asellus littoral**	**Chironomid littoral**	**Bulk_BMI profundal**	**Chironomid Profundal**	**Ruffe**	**Vendace**	**Smelt**	**Bleak**	**Roach small**	**Roach large**	**Perch small**	**Perch medium**	**Perch large**	**Pike**	**Sum**
Alajärvi	2		2		2	2	2	1	1	5	5		4	5	5	5	7	2		**50**
Arkusjärvi	2		1	1	2	2		1	1	5		5	5	5	5	5	5	5	2	**52**
Eräjärvi	2	1	1	1	2	2	1	1	1	5		5	5	5	5	3	5	5	1	**51**
Haapajärvi	2	1	1	1	2	2	2	1	1	5	2	5	5	5	5	5	5	5	2	**57**
Hiidenvesi	2	1	1	1	2	1	2	1	1	5	5	5	5	4	5	5	5	5	2	**58**
Hirvijärvi	2	1			2	2	2	1	1	5					5	3	5	5	5	**39**
Horkajärvi	2	1	1	1	2	2	2										1	2		**14**
Hämeenjärvi	2		1	1	2	2		1	1	5	2	5	5	1	5	4	6	5	2	**50**
Joroisselkä	2	1	1	1	2	2	1	1	1	5	5	5	5	1	6	5	5	5	5	**59**
Jyväsjärvi	17	7	14	12	11	6	4	12	9	5		3	5		5	5	6	5	4	**130**
Kakkisenjärvi	2		2	1	2	1			1	5				1	5	1	6	4		**31**
Kakskerranjärvi	2	1	1	1	2	2		1	1	5		5		3	5	4	5	5	1	**44**
Kuhajärvi	3		2	2	2	2	2	1	1	5				5	5	5	5	5	2	**47**
Kuontijärvi	2		1	1	2	2	2	1	1						5	3	5	3	4	**32**
Köyliönjärvi	2		1	1	2	2	2	1	1	5		5	3	5	7	7	5	5	2	**56**
Majajärvi	2		1	1	2	2	2									3		3	1	**17**
Niemisjärvi	2				2	2	2	1	1	5			1	5	4	5	5	5	3	**43**
Pankajärvi	2	1		1	2	1	2	1	1	5			5	2	5	4	5	4	4	**45**
Pesosjärvi	1		5	2	2		2	1	1								5	5		**24**
Pusulanjärvi	2	1	2	2	2	1	1	1	1	5		5	5	5	5	5	5	5	1	**54**
Pyhäjärvi	1		1	2	2	1		1		5		5	5	5	5	5	5	5	2	**50**
Pääjärvi	8		2	5	6	2	4	6	1	5	3	1			5	5	5	5		**63**
Ruokojärvi	2	1	1	1	2	1		1	1	5		5	1	4	6	1	5	5	1	**43**
Suuri-Vahvanen	2		1		2	2	1	1	1	5		5	6	5	5	5	5	5		**51**
Suuri Jukajärvi	2		1		2	2	2	1	1	4	1	5	4		6	3	6	5		**45**
Sääksjärvi	18		8	8	14	14	12	7	12	5				5	5	4	5	5	1	**123**
Sääskjärvi	2	1	1	1	2	2	1	1	1	5		5	5	5	5	4	5	4	1	**51**
Tottijärvi	2		2	2	2	1	2	1	1	5			3	5	5	5	5	5		**46**
Valkea-Kotinen	2	1			2	2	2		1								6		2	**18**
Vesijärvi			2		1	1	1	1	1	5	1	5	5	3	7		5	5		**43**
Viippero	2	1	2	2	2	1	2		1	5				1	5	4	6	5	1	**40**
Viitaanjärvi	2	1	1	1	2	1	2	1	1	5	5	5	5	5	5	6	5	5	3	**61**
Villikkalanjärvi	2		1	2	2	2	2	1		5		4	5	5	4	5	5	5	2	**52**
Ylisjärvi	2		1	1	2	2	1	1	1	5		5	5	6	5	3	6	5	1	**52**
Älänne	1	1	1	1	2	2		1	1	5	5		5		5	5	4	5	2	**46**
**Sum**	**103**	**22**	**63**	**57**	**94**	**74**	**63**	**52**	**50**	**149**	**34**	**93**	**102**	**101**	**160**	**132**	**174**	**157**	**57**	**1737**

Supplementary Table 5. Summary table of the sample sizes for each consumer group.

Group	mean	SD	Median	Min	Max	Total
**Fish**	**4.4**	**1.4**	**5**	**1**	**7**	**1159**
Benthivores	5.0	0.2	5	4	5	149
Generalists	4.8	1.2	5	1	7	435
Planktivores	4.3	1.3	5	1	7	361
Piscivores	3.6	1.6	4	1	5	214
**Invertebrates**	**2.2**	**2.6**	**2**	**1**	**18**	**578**
Zooplankton	2.2	2.9	1	1	18	245
Littoral zoobenthos	2.4	2.3	2	1	14	231
Profundal zoobenthos	1.6	2.4	1	1	12	102

Supplementary Table 6. Model slope and intercept posterior results. Median (and 95% credibility intervals) ILR-space (isometric log-ratio scale) slope, ILR-space intercept and p-space (proportional space) intercept for the different consumer taxa**.** Positive ILR slope values indicate negative connection with PC1 and consumer terrestrial resource utilization. Lower ILR intercept values indicate higher terrestrial and lower aquatic dietary utilization (taken at PC1=0, where species intercepts are offset from the global intercept). Proportional scale intercepts are shown only for terrestrial resource source. P space intercept indicates taxon-specific terrestrial dietary contribution in an average environment i.e. when PC1 is 0. Source data are provided as a Source Data file.

Taxon	ILR slope (ilr.cont.r)	ILR intercept (ilr.fac1)	p space intercept (p.fac1)
Global	0.84 (0.52 – 1.20)	1.39 (0.51 – 2.21)	0.12 (0.04 – 0.32)
**Zooplankton**			
Bulk_zooplankton	0.64 (0.34 – 0.97)	0.13 (-0.68 – 1.07)	0.11 (0.06 – 0.16)
Chaoborus	1.32 (0.36 – 2.13)	1.78 (0.66 – 3.63)	0.01 (0.00 – 0.04)
Cladocera	0.35 (0.09 – 0.68)	-0.51 (-1.32 – 0.36)	0.23 (0.15 – 0.30)
Copepods	0.43 (-0.34 – 1.28)	2.51 (0.92 – 5.21)	0.00 (0.00 – 0.03)
**Littoral zoobenthos**			
Bulk lit. zoobenthos	0.13 (-0.06 – 0.33)	-1.19 (-1.98 – -0.32)	0.43 (0.35 – 0.50)
Asellus	0.04 (-0.17 – 0.24)	-1.65 (-2.44 – -0.80)	0.59 (0.52 – 0.66)
Chironomid	0.72 (0.45 – 1.08)	-0.82 (-1.61 – 0.07)	0.31 (0.21 – 0.40)
**Profundal zoobenthos**			
Bulk prof. zoobenthos	0.40 (0.15 – 0.71)	-1.94 (-2.75 – -1.06)	0.68 (0.60 – 0.76)
Chironomid	0.68 (0.38 – 1.04)	-2.55 (-3.36 – -1.68)	0.84 (0.77 – 0.89)
**Piscivores**			
Perch (>150 mm)	1.22 (0.89 – 1.60)	-0.16 (-0.96 – 0.75)	0.15 (0.09 – 0.22)
Pike	0.94 (0.54 – 1.43)	-0.47 (-1.28 – 0.47)	0.21 (0.13 – 0.32)
**Planktivores**			
Perch (<70 mm)	1.35 (0.91 – 1.90)	1.52 (0.58 – 2.65)	0.02 (0.00 – 0.04)
Smelt	0.96 (-0.01 – 2.01)	2.68 (1.32 – 5.01)	0.00 (0.00 – 0.02)
Vendace	1.79 (1.06 – 2.69)	1.36 (0.24 – 2.84)	0.02 (0.00 – 0.07)
Bleak	1.13 (0.72 – 1.59)	0.30 (-0.52 – 1.22)	0.08 (0.04 – 0.14)
**Generalists**			
Perch (70–150 mm)	1.14 (0.82 – 1.49)	0.47 (-0.33 – 1.38)	0.07 (0.04 – 0.11)
Roach (>100 mm)	1.02 (0.68 – 1.40)	0.61 (-0.23 – 1.56)	0.06 (0.03 – 0.10)
Roach (<100 mm)	1.13 (0.43 – 2.03)	1.22 (0.19 – 2.65)	0.03 (0.00 – 0.06)
**Benthivores**			
Ruffe	0.50 (0.28 – 0.78)	-0.67 (-1.47 – 0.18)	0.27 (0.20 – 0.34)

Supplementary Table 7. Fish stomach content data (prey proportions in %). Fish species are organized based on feeding guilds (piscivores, planktivores, generalists, benthivores). Source data are provided as a Source Data file.

	Prey proportions (%)					
**Fish taxa**	Algae/Aq. Plant	Zooplankton	Benthos	Fish	Terrestrial	Detritus/Other
Pike	0.0	0.0	0.0	100.0	0.0	0.0
Perch >150 mm	0.0	7.3	11.7	72.0	0.0	8.9
Perch <70 mm	0.0	96.7	1.1	0.0	0.0	2.2
Bleak	4.1	27.2	6.6	0.0	6.3	55.8
Smelt	0.0	84.4	8.9	0.0	0.0	6.7
Vendace	0.0	53.1	15.1	0.0	13.3	18.6
Perch 70–150 mm	0.0	53.7	22.1	17.5	0.1	6.6
Roach <100 mm	1.4	28.9	34.1	0.0	0.0	35.6
Roach >100 mm	8.6	23.0	10.6	0.0	0.8	57.1
Ruffe	0.1	8.1	79.3	1.4	0.0	11.1

Supplementary Table 8. Comparison of MixSIAR models with differing model structures. Deviance information criterion (DIC), leave-one-out cross-validation information criterion (LOOic, plus standard error, SE), LOOic difference and the corresponding standard error are resented in the following columns. Relative model support is given as Akaike weights of LOOic values that sum to one across nested models. Mean stretch errors (Multiplicative error term, ξ_H) for hydrogen isotope (standard deviation in parentheses) are presented in addition to a posterior approximation of the proportional isotope variance explained by the model (approximate R^2). Based on LOOic and their standard errors, M1 and M2 performed equivalently well and were significantly better than models lacking a continuous covariate. Based on DIC and approximate R^2, model with PC1 (M1) as continuous variable performed the best. Source data available through the linked GitHub repository.

Model	DIC	LOOic	se_LOOic	dLOOic	se_dLOOic	weight	ξ_H (SD)	Approximate R^2
M1	2384.4	2159.4	80.8	6.1	21.1	0.045	0.467 (0.024)	0.379 (0.363 - 0.394)
M2	2417.1	2153.3	82.3	0	NA	0.955	0.467 (0.024)	0.364 (0.347 - 0.380)
M3	2418.2	2174.7	83.4	21.4	19.3	0	0.469 (0.024)	0.363 (0.345 - 0.379)
M4	2501.7	2227.9	82.4	74.6	27.6	0	0.497 (0.026)	0.337 (0.318 - 0.354)
M5	3327.7	3272.7	72.2	1119.4	71.3	0	0.788 (0.044)	0.110 (0.076 - 0.139)
M6[a]	14224.3	3667.9	56.1	1514.6	77.7	0	1.046 (0.048)	-

M1: 1 + PC1 + (1+PC1|species) +(1|Lake), **M2**: 1 + POM N:C +(1+POM N:C |species) +(1|Lake), **M3**: 1 + Forest % + (1+Forest %|species) + (1|Lake), **M4:** 1 + (1|species) +(1|Lake), **M5:** 1 + (1|Lake), **M6:** 1. [a] approximate R^2 is not appropriate for the intercept only model as there is no attribution of isotope variation to other variables.

Supplementary Table 9. Recalculations of hydrogen isotope omega (ω) values based on the suggestion by Soto et al. (2017, Table 2 equation a) to recalibrate δ^2H value of consumers due to bias in earlier standard values. The left side of the table (dashed vertical line) summarizes omega values provided in the best available review paper (Brett et al., 2018). On the right side of the dashed line, we have listed the standards used for δ^2H analyses of pulverized sample powder. ω calc. method = omega value calculation method where Arithmetic refers to formula: $\omega = (\Delta\delta^2H_{\text{consumer}})/(\Delta\delta^2H_{\text{environmental water}})$ and linear model to linear regression slope in model $\delta^2H_{\text{consumer}} \sim \delta^2H_{\text{water}}$. $\omega_{\text{recalc.}}$ is recalculated omega values with the uncorrected data that were available, if multiple samples were available we used mean of the samples. $\omega_{\text{d2H cor. \& recalc.}}$ = corrected omega values with δ^2H corrected consumer values. Δω express the difference between ω values reported by Brett et al. (2018) and the δ^2H corrected omega values ($\omega_{\text{d2H cor. \& recalc}}$). As we did not have the access to actual data of most of the studies listed in the table, we retrieved the data in many cases from the figures. Source data available through linked publications.

Study	Organism	Mean	SD	used standards	ω calc. method	$\omega_{\text{recalc.}}$	$\omega_{\text{d2H cor. \& recalc.}}$	Δω
[a]M. W. O'Neill et al., unpublished data†	trout	0.23	0.03	NA	NA	NA	NA	NA
[b]Macko et al. (1983)	marine amphipod	0.12		NA	NA	NA	(0.12)	NA
*Solomon et al. (2009)	Daphnia	0.20	0.04	CFS, CHS, BWB	Arithmetic	0.206*	0.175*	-0.030*
*Solomon et al. (2009)	mosquito larva	0.39	0.04	CFS, CHS, BWB	Arithmetic	0.291*	0.248*	-0.043*
*Solomon et al. (2009)	salmonid fish	0.12	0.02	CFS, CHS, BWB	linear model	0.129*	0.110*	-0.019*
Wang et al. (2009)	chironomids	0.31	0.03	BWB	linear model	NA	0.267	-0.043
Soto et al. (2013)	chironomids	0.47	0.04	CBS, KHS	linear model	NA	0.416	-0.054
Soto et al. (2013)	guppy	0.33	0.003	CBS, KHS	linear model	NA	0.283	-0.047
[c]Graham et al. (2014)	Atlantic salmon	0.36	0.05	CBS, KHS	Arithmetic	NA	NA	NA
[c]Graham et al. (2014)	Artic charr	0.35	0.05	CBS, KHS	Arithmetic	NA	NA	NA
[d]Hondula and Pace (2014)	estuarine clams	0.15	0.09	CFS, CHS, BWB	NA	NA	NA	NA
[e]Newsome et al. (2017)	Nile tilapia[f]	0.24	0.02	Several	NA	NA	0.231[g]	NA
Group average		0.27	0.11				(0.231±0.089)	-0.039

[a] No access to this study. [b] δ^2H analyzed with the similar method as water. [c] This study did not estimate ω with water labelling experiments, thus it was excluded from the recalculations of omega. [d] No water, food or organism values presented and thus this study was excluded from recalculations of omega. [e] Several standards, correct values. [f] Mean of Nile tilapia muscle and liver omega values. [g] Value for only Nile tilapia muscle. * Values for consumers δ^2H are copied from fig. 1-2 (Solomon et al., 2009), also the calculation of omega included bootstrapping, thus we recalculated the omega values with best we could and the omega value differs from their original report, therefore here Δω refers to the difference between $\omega_{\text{d2H cor. \& recalc.}}$ and ω_{recalc}.

Macko, S. A., M. L. F. Estep, and W. Y. Lee. 1983. Stable hydrogen isotope analysis of food webs on laboratory and field populations of marine amphipods. Journal of Experimental Marine Biology and Ecology 72:243–249.

Solomon, C. T., J. J. Cole, R. R. Doucett, M. L. Pace, N. D. Preston, L. E. Smith, and B. C. Weidel. 2009. The influence of environ-mental water on the hydrogen stable isotope ratio in aquatic consumers. Oecologia 161:313–324.

Wang, Y. V., D. M. O'Brien, J. Jenson, D. Francis, and M. J. Wooller. 2009. The influence of diet and water on the stable oxygen andhydrogen isotope composition of Chironomidae (Diptera) with paleoecological implications. Oecologia 160:225–233.

Soto, D. X., L. I. Wassenaar, and K. A. Hobson. 2013. Stable hydrogen and oxygen isotopes in aquatic food webs are tracers of diet and provenance. Functional Ecology 27:535–543.

Graham, C. T., S. S. C. Harrison, and C. Harrod. 2014. Differences in the contributions of environmental water to the hydrogen stable isotope ratios of cultured Atlantic salmon and Arctic charr tissues. Hydrobiologia 721:45–55.

Hondula, K. L., and M. L. Pace. 2014. Macroalgal support of cultured hard clams in a low nitrogen coastal lagoon. Marine Ecology Progress Series 498:187–201

Newsome, S. D., N. Wolf, C. J. Bradley, and M. L. Fogel. 2017. Assimilation and isotopic discrimination of hydrogen in tilapia: implications for studying animal diet with d^2H. Ecosphere 8:e01616.

Supplementary Table 10. Average summertime inlet DOM and POM δ^2H values of the studied lakes. $R^2 = 0.35$, $DOM_{d2H} = 0.9539*POM_{d2H} - 8.6506$. Source data are provided as a Source Data file.

	δ^2H (mean)	1 SD	n
inlet DOM	−120.5	9.0	35
inlet POM	−123.7	14.5	35

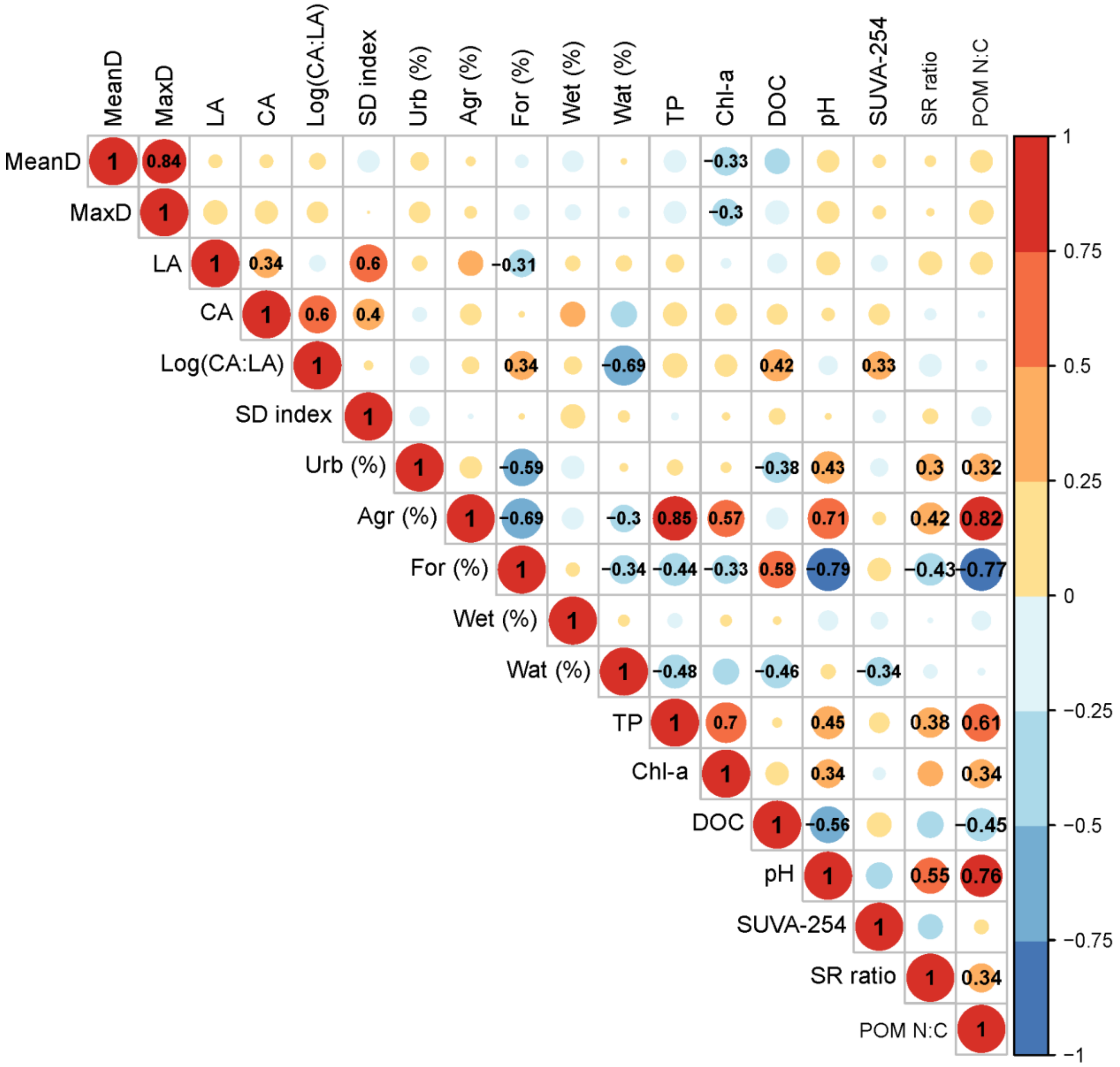

Supplementary Fig. 1. Pairwise correlations between the lake environmental variables (lake chemistry, catchment area characteristics, lake morphometrics and light measurements). Direction of correlation is indicated with colours from blue to red (negative to positive, respectively). Pearson correlation values are presented if the correlation was statistically significant (α-level 0.05), with the size of the circle presenting the strength of the correlation.

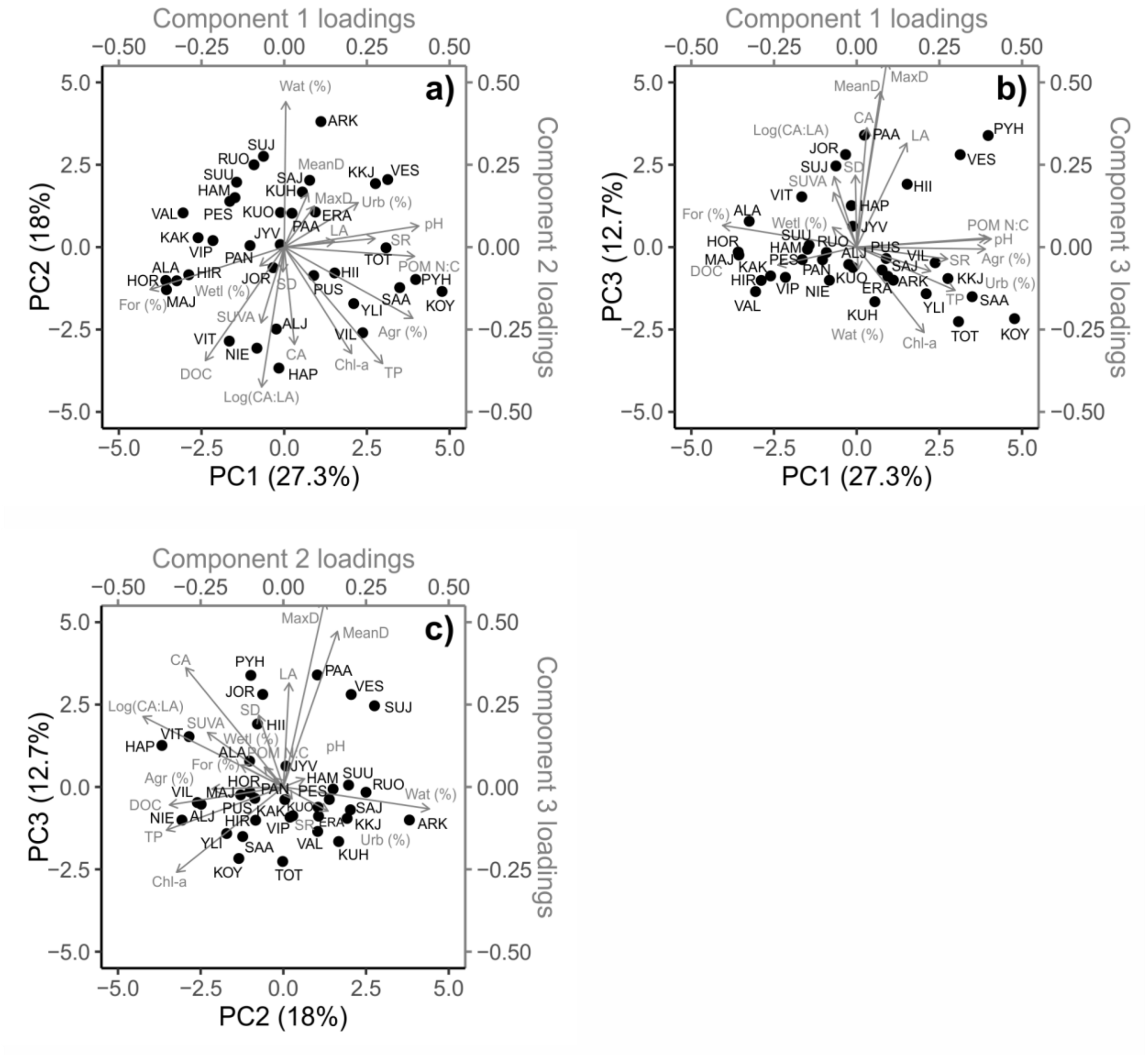

Supplementary Fig. 2. Principal component analysis ordinations of PC1 vs PC2 (a), PC1 vs PC3 and PC2 vs PC3. Environmental variable loadings are drawn with grey arrows. See Table S1 for abbreviations of the lake names, environmental variables, and PC scores. Source data are provided as a Source Data file.

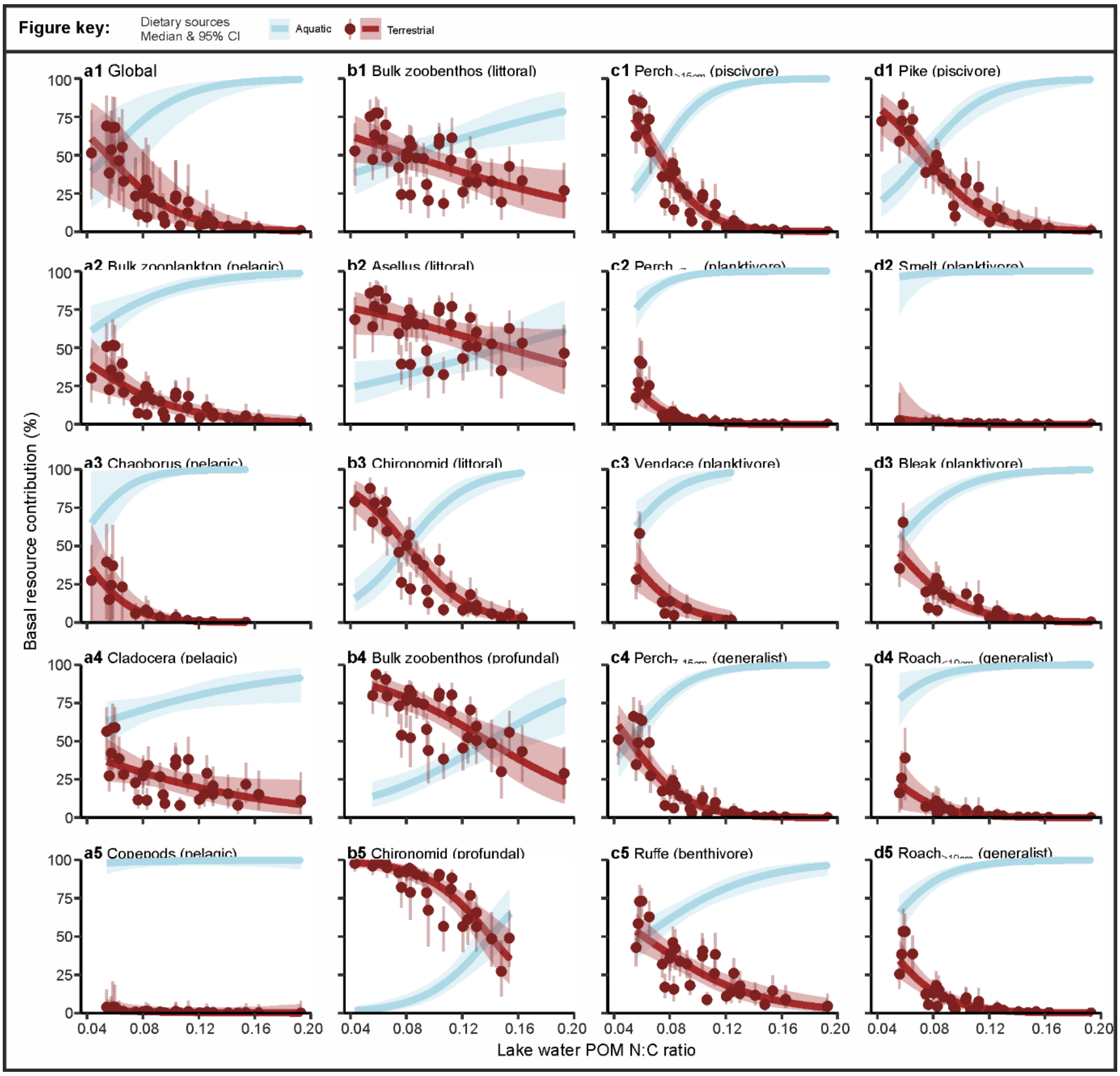

Supplementary Fig. 3. Effect of POM N:C-ratio on consumer allochthony in 35 study lakes. Data represents posterior estimates of consumer resource use derived from MixSIAR model. The bold colored lines and the light ribbon areas indicate the median and 95% credible intervals, respectively, of the Bayesian estimates of consumer resource use across lake POM N:C ratio gradient, low POM N:C ratios values indicate more terrestrial material and higher values more autochthonous material in lake POM[29,49]. Lake- and consumer-specific estimates of allochthony are marked with brown dots and shaded lines, respectively. Zooplankton (a), zoobenthos (b), and fish consumers (c-d) are divided into different columns. Consumer group names are presented for each subplot in the corresponding header, where text inside the brackets indicate habitats (a-b) or feeding guilds (c-d). Source data are provided as a Source Data file.

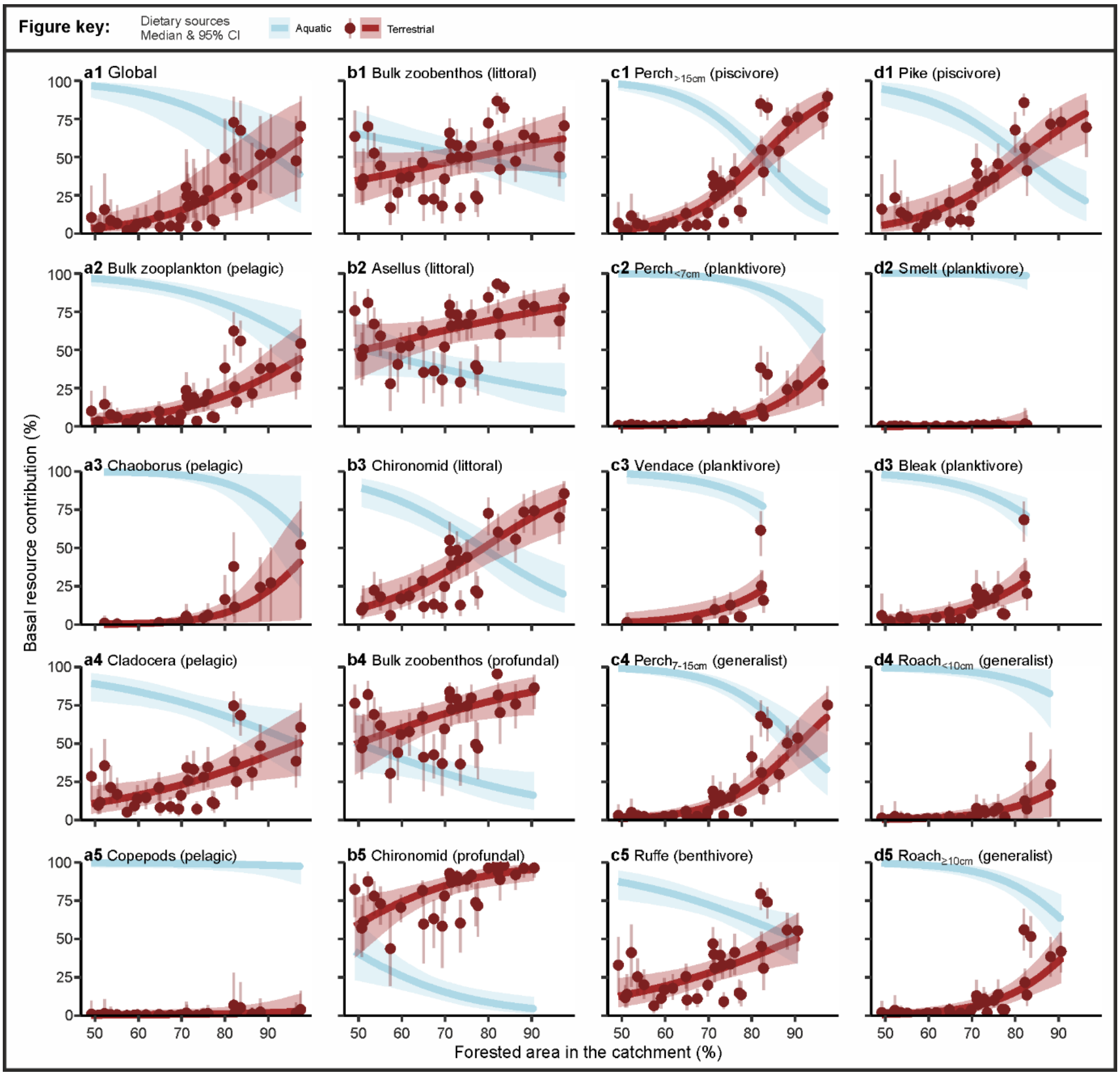

Supplementary Fig. 4. Effect of forest coverage on consumer allochthony in 35 study lakes. Data represents posterior estimates of consumer resource use derived from MixSIAR model. The bold colored lines and the light ribbon areas indicate the median and 95% credible intervals, respectively, of the Bayesian estimates of consumer resource use across lake catchment forest coverage (%) gradient. Lake- and consumer-specific estimates of allochthony are marked with brown dots and shaded lines, respectively. Zooplankton (a), zoobenthos (b), and fish consumers (c-d) are divided into different columns. Consumer group names are presented for each subplot in the corresponding header, where text inside the brackets indicate habitats (a-b) or feeding guilds (c-d). Source data are provided as a Source Data file.

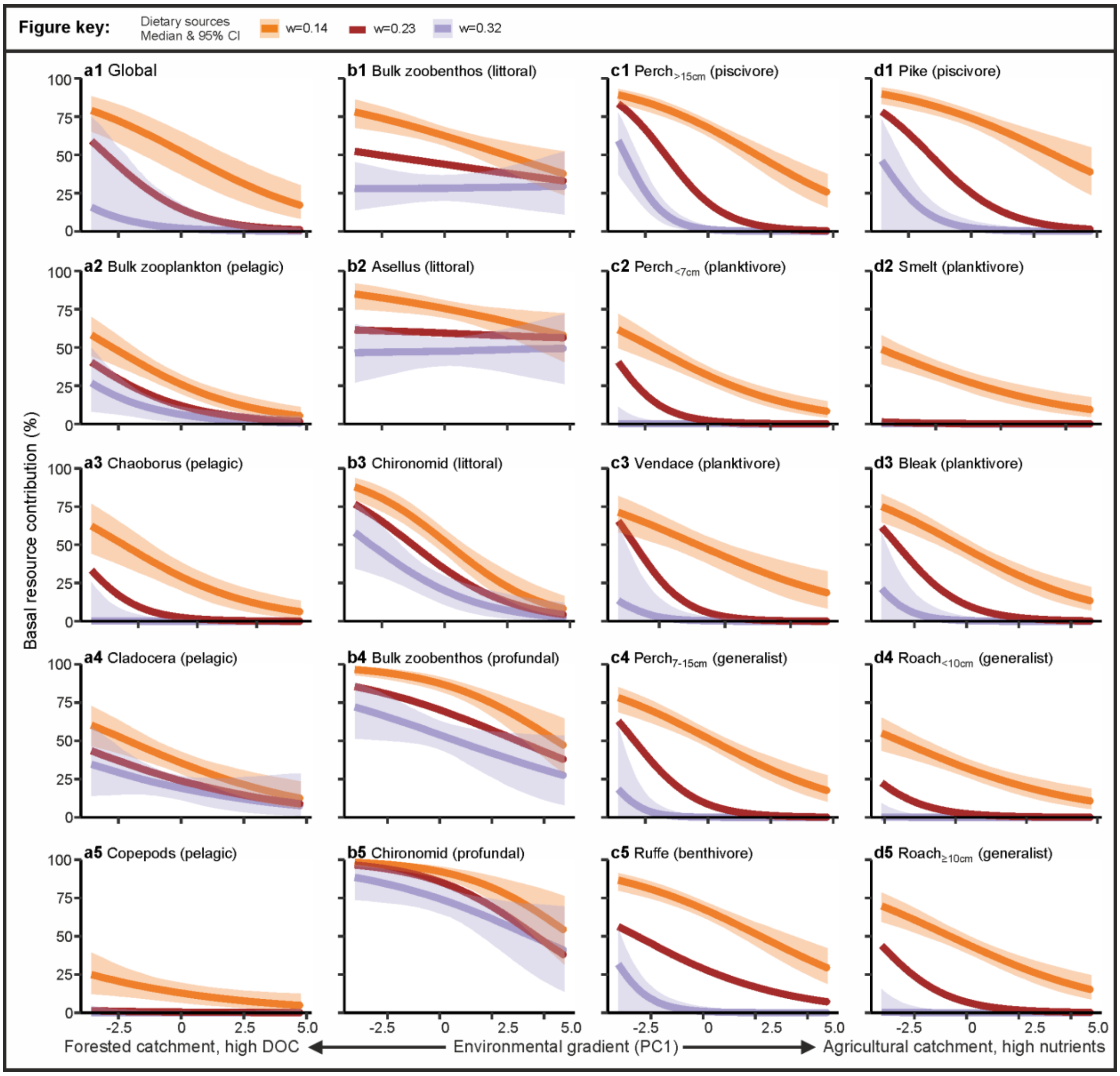

Supplementary Fig. 5. Omega sensitivity analysis for MixSIAR mixing models. Allochthony of consumers is estimated using omega values of 0.14, 0.23 and 0.33 marked with orange, brown and purple lines, respectively, with the lines and shaded ribbon areas indicating the median and 95% credible intervals of the Bayesian estimates. Zooplankton (a), zoobenthos (b), and fish consumers (c-d) are divided into different columns. Consumer group names are presented for each subplot in the corresponding header, where the text inside the brackets indicate habitats (a-b) or fish feeding guilds (c-d). Source data are provided as a Source Data file.

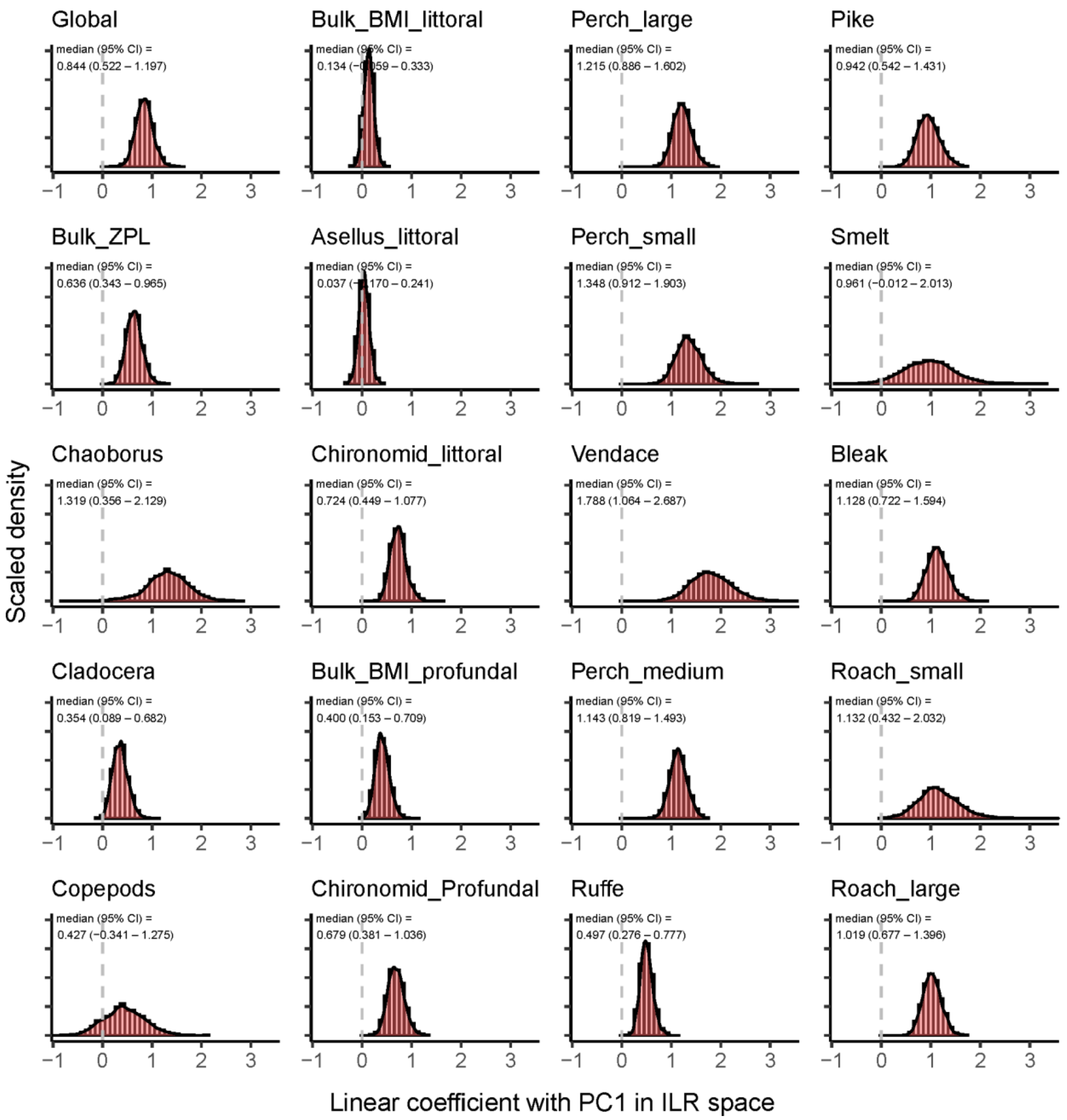

Supplementary Fig. 6. Scaled density of linear regression coefficient with PC1 and dietary mixtures in ILR space. Median and 95% Bayesian Credible Intervals (CI) of the coefficient are shown in the figure for each consumer group and the global posterior estimate. When coefficient distribution is non-zero it indicates that PC1 had significant effect on consumer allochthony since with our model configuration ILR space is one dimensional. Positive ILR slope values indicate negative PC1 connection with consumer terrestrial resource utilization as MixSIAR package load_source_data() function organizes sources alphabetically. For further information and toy data example, see GitHub repository “1. Scripts/4. model comparisons test.R”. Source data are provided as a Source Data file.

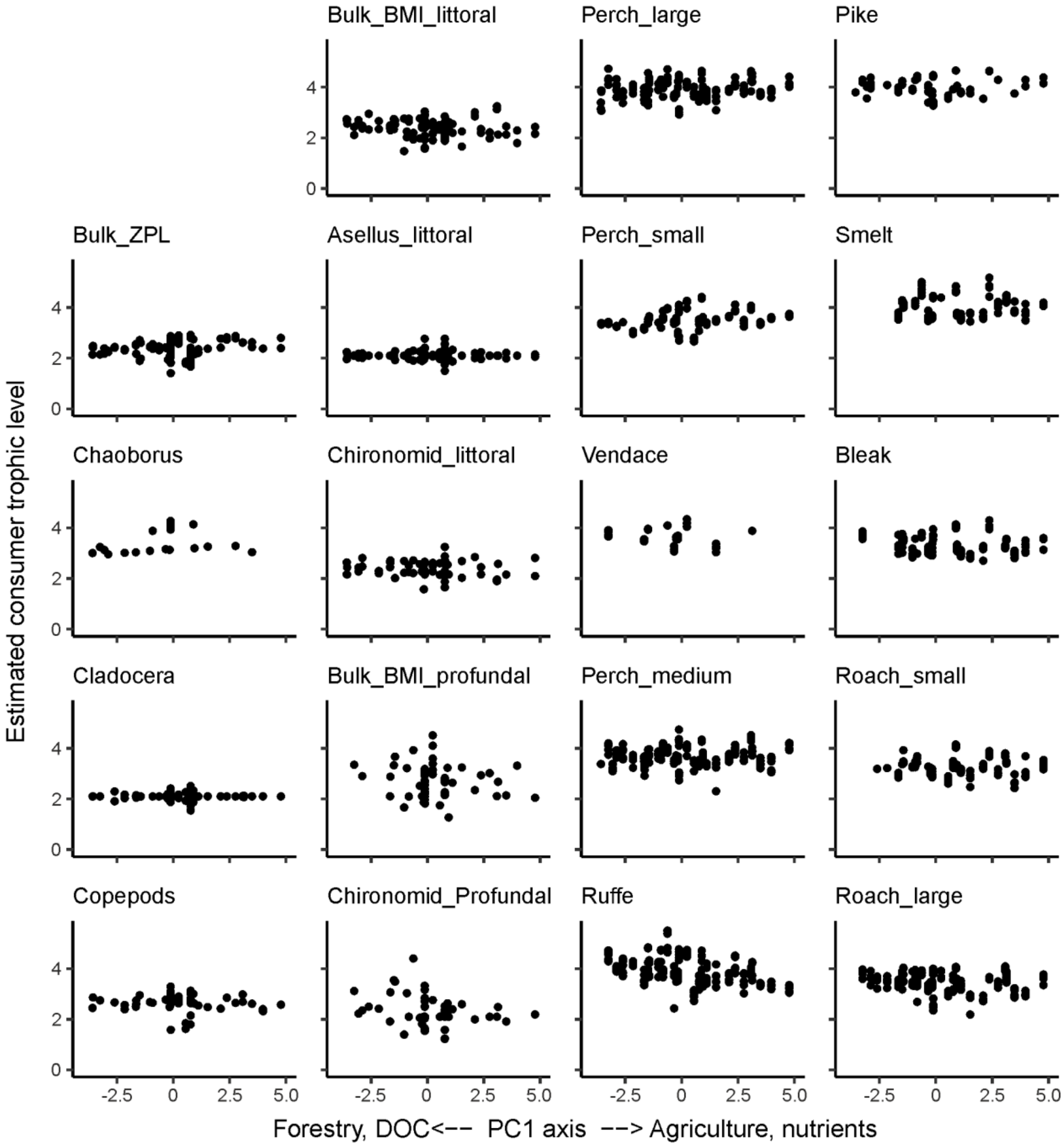

Supplementary Fig. 7. Estimated consumer trophic level across the environmental gradient (PC1). Black dots represent estimated consumer trophic levels that were used in calculating the $\omega_{compound}$. Based on visual inspection of these data, and the lack of clear positive trends between the PC1 and Trophic level, it is obvious that the trophic level estimations are not driving the observed decreasing consumer allochthony patterns across the studied environmental gradient (PC1). Source data are provided as a Source Data file.

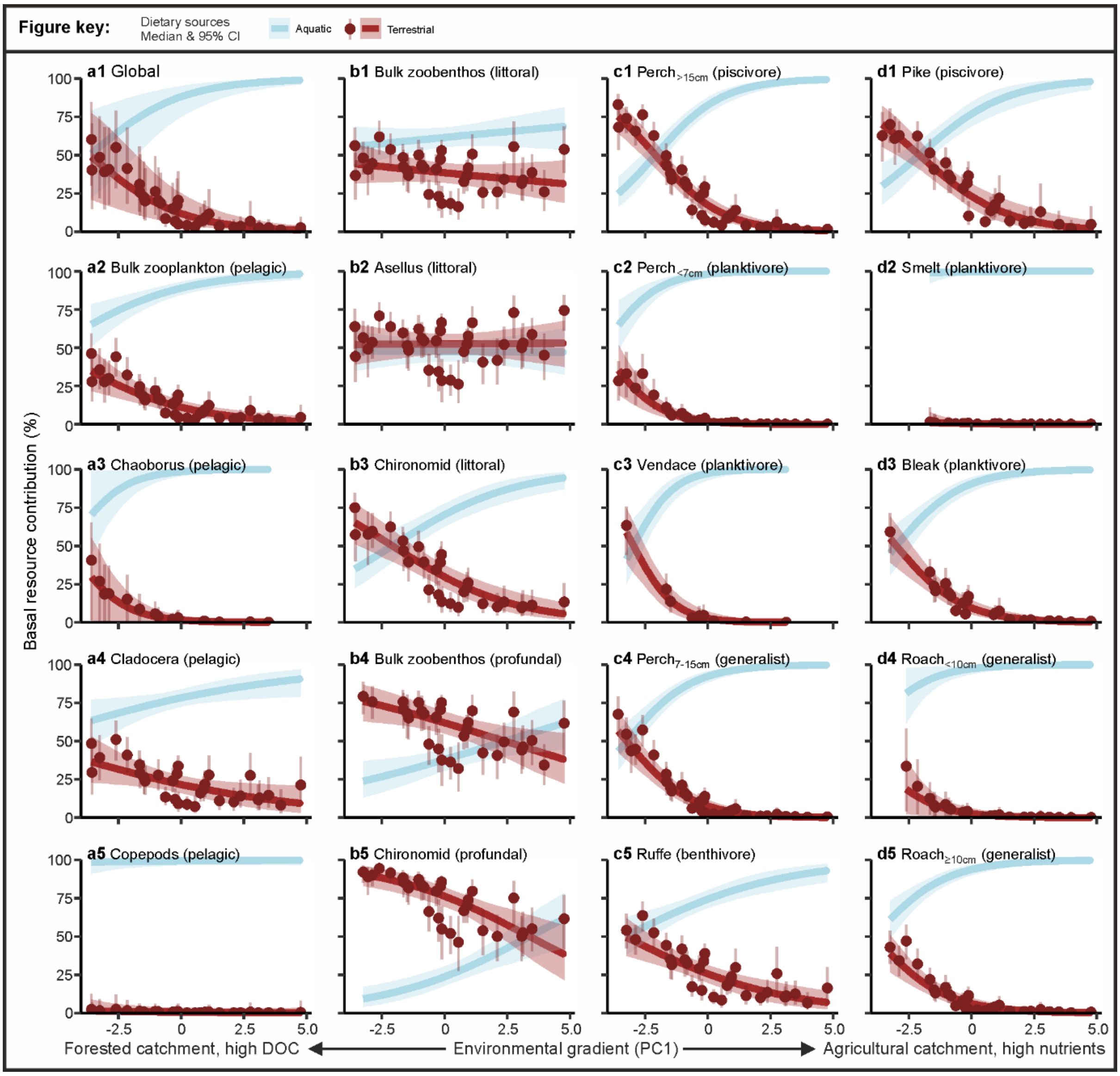

Supplementary Fig. 8. Consumer allochthony models with modelled bacterial δ²H values as terrestrial source. Modelled bacterial δ²H values were obtained with following equation: ($\delta^2H_{bact} = \delta^2H_{inlet\ DOM} \times (1 - \omega) + \delta^2H_{lake\ water} \times \omega$) where the used omega value was 0.17. The modelled phytoplankton values were used as aquatic source for the consumers. The bold colored lines and the light ribbon areas indicate the median and 95% credible intervals, respectively, of the Bayesian estimates of consumer resource use across lake catchment forest coverage (%) gradient. Lake- and consumer-specific estimates of allochthony are marked with brown dots and shaded lines, respectively. Zooplankton (a), zoobenthos (b), and fish consumers (c-d) are divided into different columns. Consumer group names are presented for each subplot in the corresponding header, where text inside the brackets indicate habitats (a-b) or feeding guilds (c-d). Source data are provided as a Source Data file.

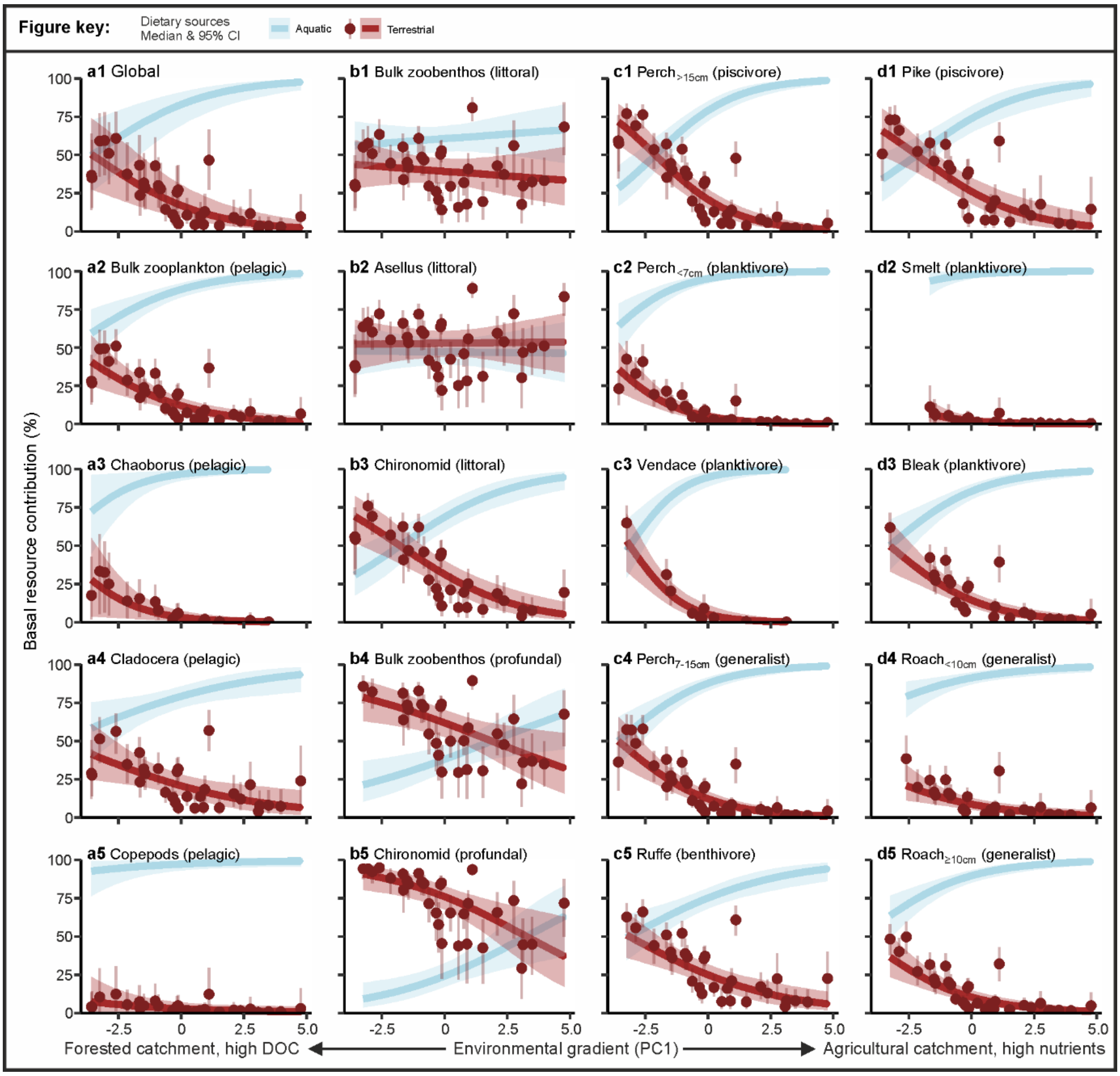

Supplementary Fig. 9. Consumer allochthony models with sampled benthic algae δ²H values as aquatic source. The bold colored lines and the light ribbon areas indicate the median and 95% credible intervals, respectively, of the Bayesian estimates of consumer resource use across lake catchment forest coverage (%) gradient. Lake- and consumer-specific estimates of allochthony are marked with brown dots and shaded lines, respectively. Zooplankton (a), zoobenthos (b), and fish consumers (c-d) are divided into different columns. Consumer group names are presented for each subplot in the corresponding header, where text inside the brackets indicate habitats (a-b) or feeding guilds (c-d). Source data are provided as a Source Data file.
