# Supplementary material for: The role of land use in terrestrial support of boreal lake food webs
Source: Nat Commun. 2025 Apr 15;16:3572. doi: 10.1038/s41467-025-58505-y (PMC12000567; doi:10.1038/s41467-025-58505-y)
Supplement: Supplementary file 2 — Reporting Summary [file 41467_2025_58505_MOESM2_ESM.pdf]

nature portfolio

Corresponding author(s): Ossi Keva, ossi.keva@helsinki.fi

Last updated by author(s): Feb 28, 2025

Reporting Summary

Nature Portfolio wishes to improve the reproducibility of the work that we publish. This form provides structure for consistency and transparency in reporting. For further information on Nature Portfolio policies, see our Editorial Policies and the Editorial Policy Checklist.

Statistics

For all statistical analyses, confirm that the following items are present in the figure legend, table legend, main text, or Methods section.

n/a	Confirmed	
☐	☒	The exact sample size (*n*) for each experimental group/condition, given as a discrete number and unit of measurement
☐	☒	A statement on whether measurements were taken from distinct samples or whether the same sample was measured repeatedly
☐	☒	The statistical test(s) used AND whether they are one- or two-sided<br>*Only common tests should be described solely by name; describe more complex techniques in the Methods section.*
☐	☒	A description of all covariates tested
☐	☒	A description of any assumptions or corrections, such as tests of normality and adjustment for multiple comparisons
☐	☒	A full description of the statistical parameters including central tendency (e.g. means) or other basic estimates (e.g. regression coefficient) AND variation (e.g. standard deviation) or associated estimates of uncertainty (e.g. confidence intervals)
☒	☐	For null hypothesis testing, the test statistic (e.g. *F*, *t*, *r*) with confidence intervals, effect sizes, degrees of freedom and *P* value noted<br>*Give P values as exact values whenever suitable.*
☐	☒	For Bayesian analysis, information on the choice of priors and Markov chain Monte Carlo settings
☐	☒	For hierarchical and complex designs, identification of the appropriate level for tests and full reporting of outcomes
☐	☒	Estimates of effect sizes (e.g. Cohen's *d*, Pearson's *r*), indicating how they were calculated

Our web collection on statistics for biologists contains articles on many of the points above.

Software and code

Policy information about availability of computer code

Data collection	Water chemistry data is available at HERTTA open database maintained by the Finnish Environment Institute (syke.fi/avointieto). Corine land use level 4 data is available from Finnish Environment Institute (syke.fi/avointieto). Geospatial shape files used for map producing are available through Natural Earth (naturalearthdata.com)
Data analysis	We used the MixSIAR package (version 3.1.12) in R (version 4.2.1) to estimate terrestrial basal resource use of consumers (consumer allochthony) in each lake. R scripts used to consumer allochthony modelling are available at a public Github repository (osjomike/Keva-et-al.-2025-NC ; https://doi.org/10.5281/zenodo.14792582), where all the needed R packages with version numbers are presented.<br>Data arrangement: plyr (version: 1.1.4), dplyr (1.1.0), reshape2 (1.1.4)<br>Graphics: ggplot2 (3.4.1), ggnewscale (0.5.0), cowplot (1.1.3), gridExtra (2.3), splancs(2.01-45), ggrepel (0.9.6), ggpubr (0.6.0)<br>MixSIAR stuff: MixSIAR (3.1.12), rjags (4-16), R2WinBUGS (2.1-22.1)<br>Just Another Gibbs Sampler JAGS (version 4.3.0.)

For manuscripts utilizing custom algorithms or software that are central to the research but not yet described in published literature, software must be made available to editors and reviewers. We strongly encourage code deposition in a community repository (e.g. GitHub). See the Nature Portfolio guidelines for submitting code & software for further information.

Data

Policy information about availability of data

All manuscripts must include a data availability statement. This statement should provide the following information, where applicable:

- Accession codes, unique identifiers, or web links for publicly available datasets
- A description of any restrictions on data availability
- For clinical datasets or third party data, please ensure that the statement adheres to our policy

All datasets used in this study are publicly available. Geospatial shape files used for map producing are available through Natural Earth (naturalearthdata.com). Water chemistry data are available at the HERTTA open database maintained by the Finnish Environment Institute (www.syke.fi/avointieto). Corine land use level 4 data are available from the Finnish Environment Institute (syke.fi/avointieto). A compiled version of these environmental variables is presented in Supplementary Table 1. The stable isotope data are available through a GitHub repository [https://doi.org/10.5281/zenodo.14792582]. Source data are provided with this paper.

Research involving human participants, their data, or biological material

Policy information about studies with human participants or human data. See also policy information about sex, gender (identity/presentation), and sexual orientation and race, ethnicity and racism.

Reporting on sex and gender	*Use the terms sex (biological attribute) and gender (shaped by social and cultural circumstances) carefully in order to avoid confusing both terms. Indicate if findings apply to only one sex or gender; describe whether sex and gender were considered in study design; whether sex and/or gender was determined based on self-reporting or assigned and methods used. Provide in the source data disaggregated sex and gender data, where this information has been collected, and if consent has been obtained for sharing of individual-level data; provide overall numbers in this Reporting Summary. Please state if this information has not been collected. Report sex- and gender-based analyses where performed, justify reasons for lack of sex- and gender-based analysis.*
Reporting on race, ethnicity, or other socially relevant groupings	*Please specify the socially constructed or socially relevant categorization variable(s) used in your manuscript and explain why they were used. Please note that such variables should not be used as proxies for other socially constructed/relevant variables (for example, race or ethnicity should not be used as a proxy for socioeconomic status). Provide clear definitions of the relevant terms used, how they were provided (by the participants/respondents, the researchers, or third parties), and the method(s) used to classify people into the different categories (e.g. self-report, census or administrative data, social media data, etc.) Please provide details about how you controlled for confounding variables in your analyses.*
Population characteristics	*Describe the covariate-relevant population characteristics of the human research participants (e.g. age, genotypic information, past and current diagnosis and treatment categories). If you filled out the behavioural & social sciences study design questions and have nothing to add here, write "See above."*
Recruitment	*Describe how participants were recruited. Outline any potential self-selection bias or other biases that may be present and how these are likely to impact results.*
Ethics oversight	*Identify the organization(s) that approved the study protocol.*

Note that full information on the approval of the study protocol must also be provided in the manuscript.

Field-specific reporting

Please select the one below that is the best fit for your research. If you are not sure, read the appropriate sections before making your selection.

☐ Life sciences ☐ Behavioural & social sciences ☒ Ecological, evolutionary & environmental sciences

For a reference copy of the document with all sections, see nature.com/documents/nr-reporting-summary-flat.pdf

Ecological, evolutionary & environmental sciences study design

All studies must disclose on these points even when the disclosure is negative.

Study description	We collected a extensive biotracer dataset (stable isotopes) from 35 boreal Finnish lakes and constructed Bayesian mixing models to predict the use of exogenous, terrestrial resources (i.e., consumer allochthony) by aquatic consumers at multiple trophic levels along an environmental gradient. In the Bayesian mixing model, we set lake as a random variable and consumer taxon as a fixed factor, whereas a combined environmental gradient (PC1) was added as a continuous covariate. The study includes a total of 1737 samples of aquatic consumers and their food sources analysed for hydrogen stable isotopes.

Research sample	We sampled 19 different consumer taxa belonging to different trophic guilds in the lake food webs, and the 35 boreal study lakes covered a marked gradient in environmental characteristics, such as land use (forestry – agriculture) in the catchment. Please find the specific information about the sampled taxa and lakes in the submitted manuscript. We collected replicate or compound samples of each taxa to obtain representative data of the populations in each lake. For fish samples please find the recorded sex from the Source Data.xlsx file (Sheet: Supplementary Table 7) linked to the manuscript.
Sampling strategy	Predetermination of sample sizes: 35 was the maximum number of lakes to be reasonably sampled within three years' time frame of the project. The sample is a representative subset of boreal Finnish lakes with different types of water chemistry and catchment characteristics. The number of isotope sample replicates for producers and consumers were also optimized based on the project length, available resources, and requirements of the isotope mixing models.
Data collection	From each study lake, we sampled water, algae, inlet particulate and dissolved organic matter, multiple invertebrate and vertebrate taxa from the pelagic, profundal and littoral zones, using standard methods. Two of the authors, Dr. Mikko Kiljunen and Dr. Jos Schilder, collected the samples (fish samples in collaboration with the personnel of Natural Resources Institute Finland), analyzed them in the laboratory, and recorded the data into data files that were saved in a permanent repository.
Timing and spatial scale	The data for the study were collected from 35 lakes located in southern and central Finland (66.30—60.36 °N & 29.01—22.24 °E) and sampled once in July–August in 2016–2018. The lakes covered as many lake types with varying catchment properties as possible. A snapshot sampling of stable isotope data during the end of growing season was sufficient for the purpose of investigating among-lake and along environmental variability in consumer allochthony. Spatial-temporal sampling was conducted for three of the study lakes to obtain within lake variance in stable isotope values for the Bayesian stable isotope models.
Data exclusions	No data were excluded.
Reproducibility	All the stable isotope analyses were run following a standardized protocol: All stable isotope ratios are expressed as delta values (δ) relative to international standards (IAEA). Laboratory reference materials and working standards were analyzed after every fifth sample to check the analysis precision and to correct for potential drift during the analysis. The analytical precisions for hydrogen and nitrogen isotopes were always better than 3‰ and 0.2‰, respectively, and for water hydrogen isotopes better than 1‰. The study is based on correlative 'field experiment' but we consider the main findings robust due to the strength and consistency of the observed patterns across the environmental gradient and among consumers.
Randomization	No strict randomization was applied but the study lakes were selected from lakes belonging to a monitoring network with sufficient previous knowledge available, to guarantee representativeness of important lake gradients in the study setting. Within lakes, the samples for isotope analyses were either strictly randomized (fish samples from catches of test fishing) or were representative compound samples of spatial replicates.
Blinding	Blinding was not relevant for our study as all the observations were based on objective measurements not prone to subjective bias.

Did the study involve field work? ☒ Yes ☐ No

Field work, collection and transport

Field conditions	The field sampling was executed in predetermined time frame and samples were collected in any type of weather. The observed variables are not sensitive to weather conditions
Location	The lakes sampled cover the southern and central part of Finland (66.30—60.36 °N & 29.01—22.24 °E), with minor variation in altitude, but intentionally variable lake morphometry, water quality and catchment characteristics.
Access & import/export	During the field work, the main lake habitats (pelagic, profundal and littoral zones) were accessed with a motorboat, or in some cases the littoral habitats were accessed by car and by foot. All these activities are permitted by the 'Everybody's rights' in Finland. Fishing requiring a permission was performed by the research fishing license of Natural Resources Institute Finland (LUKE).
Disturbance	Any disturbance was avoided by actively communicating with locals present and by avoiding sampling in the close vicinity of lakeshore properties. The sampling itself did not cause any notable disturbance to biota or environment.

Reporting for specific materials, systems and methods

We require information from authors about some types of materials, experimental systems and methods used in many studies. Here, indicate whether each material, system or method listed is relevant to your study. If you are not sure if a list item applies to your research, read the appropriate section before selecting a response.

Materials & experimental systems

n/a	Involved in the study
☐	☐ Antibodies
☐	☐ Eukaryotic cell lines
☐	☐ Palaeontology and archaeology
☐	☒ Animals and other organisms
☐	☐ Clinical data
☐	☐ Dual use research of concern
☐	☐ Plants

Methods

n/a	Involved in the study
☐	☐ ChIP-seq
☐	☐ Flow cytometry
☐	☐ MRI-based neuroimaging

Antibodies

Antibodies used

Describe all antibodies used in the study; as applicable, provide supplier name, catalog number, clone name, and lot number.

Validation

Describe the validation of each primary antibody for the species and application, noting any validation statements on the manufacturer's website, relevant citations, antibody profiles in online databases, or data provided in the manuscript.

Eukaryotic cell lines

Policy information about cell lines and Sex and Gender in Research

Cell line source(s)

State the source of each cell line used and the sex of all primary cell lines and cells derived from human participants or vertebrate models.

Authentication

Describe the authentication procedures for each cell line used OR declare that none of the cell lines used were authenticated.

Mycoplasma contamination

Confirm that all cell lines tested negative for mycoplasma contamination OR describe the results of the testing for mycoplasma contamination OR declare that the cell lines were not tested for mycoplasma contamination.

Commonly misidentified lines
(See ICLAC register)

Name any commonly misidentified cell lines used in the study and provide a rationale for their use.

Palaeontology and Archaeology

Specimen provenance

Provide provenance information for specimens and describe permits that were obtained for the work (including the name of the issuing authority, the date of issue, and any identifying information). Permits should encompass collection and, where applicable, export.

Specimen deposition

Indicate where the specimens have been deposited to permit free access by other researchers.

Dating methods

If new dates are provided, describe how they were obtained (e.g. collection, storage, sample pretreatment and measurement), where they were obtained (i.e. lab name), the calibration program and the protocol for quality assurance OR state that no new dates are provided.

☐ Tick this box to confirm that the raw and calibrated dates are available in the paper or in Supplementary Information.

Ethics oversight

Identify the organization(s) that approved or provided guidance on the study protocol, OR state that no ethical approval or guidance was required and explain why not.

Note that full information on the approval of the study protocol must also be provided in the manuscript.

Animals and other research organisms

Policy information about studies involving animals; ARRIVE guidelines recommended for reporting animal research, and Sex and Gender in Research

Laboratory animals

Study did not involve laboratory animals

Wild animals

Zooplankton samples were collected with zooplankton nets; zoobenthos with Ekmann grab, kick-net and by hand picking. Fish were caught with gill nets. Invertebrates were transported in cool boxes to lab where they were identified and separated to different plastic tubes, killed with freezing as soon as the sample was ready. Fish samples were removed from nets, euthanized by cerebral concussion causing an immediate death and placed on ice during transport to the laboratory. The sampled organisms were killed in order to get material for the stable isotope analyses reported in this study.

Reporting on sex	The sex of the invertebrate samples were not collected as it was not our study focus. Fish individual sex and sexual maturity were recorded but are not shown in this research as it was not in the focus of this study, however the data is available through the source data file (Sheet: Supplementary Table 7).
Field-collected samples	The field collected samples were stored to freezer (-20 degrees) prior to freeze-drying. Stable isotope analysis were done at University of Jyväskylä (Finland)
Ethics oversight	At all stages of this study, national guidelines (FI-564/2013 & FI-487/2013) and the European Union directive (2010/63/EU) on the protection of animals used for scientific purposes were applied.<br><br>Finnish Everyone's Rights, fishing permits from local fishery boards and Finnish Centre for Economic Development, Transport and the Environment were applied in sample collection. Permission numbers from Finnish Centre for Economic Development, Transport and the Environment: POSELY:1899/5719/2008; POSELY: 1271/5712/2012; POSELY: 1946/5713-2012; POSELY: 1013/5713-2012; POSELY: 2110/5713-2002; POSELY: 1806/5715/2012; POSELY: 2370/0713-2012; POSELY: 3844/5713/2012; POSELY: 1260/5716-2012; POSELY: 1877/5713-2012; POSELY: 1979/5716/2012

Note that full information on the approval of the study protocol must also be provided in the manuscript.

Clinical data

Policy information about clinical studies
All manuscripts should comply with the ICMJEguidelines for publication of clinical research and a completedCONSORT checklist must be included with all submissions.

Clinical trial registration	*Provide the trial registration number from ClinicalTrials.gov or an equivalent agency.*
Study protocol	*Note where the full trial protocol can be accessed OR if not available, explain why.*
Data collection	*Describe the settings and locales of data collection, noting the time periods of recruitment and data collection.*
Outcomes	*Describe how you pre-defined primary and secondary outcome measures and how you assessed these measures.*

Dual use research of concern

Policy information about dual use research of concern

Hazards

Could the accidental, deliberate or reckless misuse of agents or technologies generated in the work, or the application of information presented in the manuscript, pose a threat to:

No	Yes	
☐	☐	Public health
☐	☐	National security
☐	☐	Crops and/or livestock
☐	☐	Ecosystems
☐	☐	Any other significant area

Experiments of concern

Does the work involve any of these experiments of concern:

No	Yes	
☐	☐	Demonstrate how to render a vaccine ineffective
☐	☐	Confer resistance to therapeutically useful antibiotics or antiviral agents
☐	☐	Enhance the virulence of a pathogen or render a nonpathogen virulent
☐	☐	Increase transmissibility of a pathogen
☐	☐	Alter the host range of a pathogen
☐	☐	Enable evasion of diagnostic/detection modalities
☐	☐	Enable the weaponization of a biological agent or toxin
☐	☐	Any other potentially harmful combination of experiments and agents

Plants

Seed stocks

Report on the source of all seed stocks or other plant material used. If applicable, state the seed stock centre and catalogue number. If plant specimens were collected from the field, describe the collection location, date and sampling procedures.

Novel plant genotypes

Describe the methods by which all novel plant genotypes were produced. This includes those generated by transgenic approaches, gene editing, chemical/radiation-based mutagenesis and hybridization. For transgenic lines, describe the transformation method, the number of independent lines analyzed and the generation upon which experiments were performed. For gene-edited lines, describe the editor used, the endogenous sequence targeted for editing, the targeting guide RNA sequence (if applicable) and how the editor was applied.

Authentication

Describe any authentication procedures for each seed stock used or novel genotype generated. Describe any experiments used to assess the effect of a mutation and, where applicable, how potential secondary effects (e.g. second site T-DNA insertions, mosiacism, off-target gene editing) were examined.

ChIP-seq

Data deposition

☐ Confirm that both raw and final processed data have been deposited in a public database such as GEO.

☐ Confirm that you have deposited or provided access to graph files (e.g. BED files) for the called peaks.

Data access links
May remain private before publication.

For "Initial submission" or "Revised version" documents, provide reviewer access links. For your "Final submission" document, provide a link to the deposited data.

Files in database submission

Provide a list of all files available in the database submission.

Genome browser session
(e.g. UCSC)

Provide a link to an anonymized genome browser session for "Initial submission" and "Revised version" documents only, to enable peer review. Write "no longer applicable" for "Final submission" documents.

Methodology

Replicates

Describe the experimental replicates, specifying number, type and replicate agreement.

Sequencing depth

Describe the sequencing depth for each experiment, providing the total number of reads, uniquely mapped reads, length of reads and whether they were paired- or single-end.

Antibodies

Describe the antibodies used for the ChIP-seq experiments; as applicable, provide supplier name, catalog number, clone name, and lot number.

Peak calling parameters

Specify the command line program and parameters used for read mapping and peak calling, including the ChIP, control and index files used.

Data quality

Describe the methods used to ensure data quality in full detail, including how many peaks are at FDR 5% and above 5-fold enrichment.

Software

Describe the software used to collect and analyze the ChIP-seq data. For custom code that has been deposited into a community repository, provide accession details.

Flow Cytometry

Plots

Confirm that:

☐ The axis labels state the marker and fluorochrome used (e.g. CD4-FITC).

☐ The axis scales are clearly visible. Include numbers along axes only for bottom left plot of group (a 'group' is an analysis of identical markers).

☐ All plots are contour plots with outliers or pseudocolor plots.

☐ A numerical value for number of cells or percentage (with statistics) is provided.

Methodology

Sample preparation

Describe the sample preparation, detailing the biological source of the cells and any tissue processing steps used.

Instrument

Identify the instrument used for data collection, specifying make and model number.

Software

Describe the software used to collect and analyze the flow cytometry data. For custom code that has been deposited into a community repository, provide accession details.

Cell population abundance | *Describe the abundance of the relevant cell populations within post-sort fractions, providing details on the purity of the samples and how it was determined.*

Gating strategy | *Describe the gating strategy used for all relevant experiments, specifying the preliminary FSC/SSC gates of the starting cell population, indicating where boundaries between "positive" and "negative" staining cell populations are defined.*

☐ Tick this box to confirm that a figure exemplifying the gating strategy is provided in the Supplementary Information.

Magnetic resonance imaging

Experimental design

Design type | *Indicate task or resting state; event-related or block design.*

Design specifications | *Specify the number of blocks, trials or experimental units per session and/or subject, and specify the length of each trial or block (if trials are blocked) and interval between trials.*

Behavioral performance measures | *State number and/or type of variables recorded (e.g. correct button press, response time) and what statistics were used to establish that the subjects were performing the task as expected (e.g. mean, range, and/or standard deviation across subjects).*

Acquisition

Imaging type(s) | *Specify: functional, structural, diffusion, perfusion.*

Field strength | *Specify in Tesla*

Sequence & imaging parameters | *Specify the pulse sequence type (gradient echo, spin echo, etc.), imaging type (EPI, spiral, etc.), field of view, matrix size, slice thickness, orientation and TE/TR/flip angle.*

Area of acquisition | *State whether a whole brain scan was used OR define the area of acquisition, describing how the region was determined.*

Diffusion MRI ☐ Used ☐ Not used

Preprocessing

Preprocessing software | *Provide detail on software version and revision number and on specific parameters (model/functions, brain extraction, segmentation, smoothing kernel size, etc.).*

Normalization | *If data were normalized/standardized, describe the approach(es): specify linear or non-linear and define image types used for transformation OR indicate that data were not normalized and explain rationale for lack of normalization.*

Normalization template | *Describe the template used for normalization/transformation, specifying subject space or group standardized space (e.g. original Talairach, MNI305, ICBM152) OR indicate that the data were not normalized.*

Noise and artifact removal | *Describe your procedure(s) for artifact and structured noise removal, specifying motion parameters, tissue signals and physiological signals (heart rate, respiration).*

Volume censoring | *Define your software and/or method and criteria for volume censoring, and state the extent of such censoring.*

Statistical modeling & inference

Model type and settings | *Specify type (mass univariate, multivariate, RSA, predictive, etc.) and describe essential details of the model at the first and second levels (e.g. fixed, random or mixed effects; drift or auto-correlation).*

Effect(s) tested | *Define precise effect in terms of the task or stimulus conditions instead of psychological concepts and indicate whether ANOVA or factorial designs were used.*

Specify type of analysis: ☐ Whole brain ☐ ROI-based ☐ Both

Statistic type for inference | *Specify voxel-wise or cluster-wise and report all relevant parameters for cluster-wise methods.*

(See Eklund et al. 2016)

Correction | *Describe the type of correction and how it is obtained for multiple comparisons (e.g. FWE, FDR, permutation or Monte Carlo).*

Models & analysis

n/a	Involved in the study
☐	☐ Functional and/or effective connectivity
☐	☐ Graph analysis
☐	☐ Multivariate modeling or predictive analysis

Functional and/or effective connectivity	*Report the measures of dependence used and the model details (e.g. Pearson correlation, partial correlation, mutual information).*
Graph analysis	*Report the dependent variable and connectivity measure, specifying weighted graph or binarized graph, subject- or group-level, and the global and/or node summaries used (e.g. clustering coefficient, efficiency, etc.).*
Multivariate modeling and predictive analysis	*Specify independent variables, features extraction and dimension reduction, model, training and evaluation metrics.*
